# The environmental impact of changing to virtual renal transplant aftercare: 2-year experience with a single outpatient clinic

**DOI:** 10.1016/j.fhj.2024.100004

**Published:** 2024-02-19

**Authors:** Louise Moore, Frances Balmer, Alexander Woywodt

**Affiliations:** a Department of Renal Medicine, Lancashire Teaching Hospitals NHS Foundation Trust, Preston, U.K; b Sustainability Fellow, Lancashire Teaching Hospitals NHS Foundation Trust, Preston, U.K

## Introduction

**Fig. 1:**
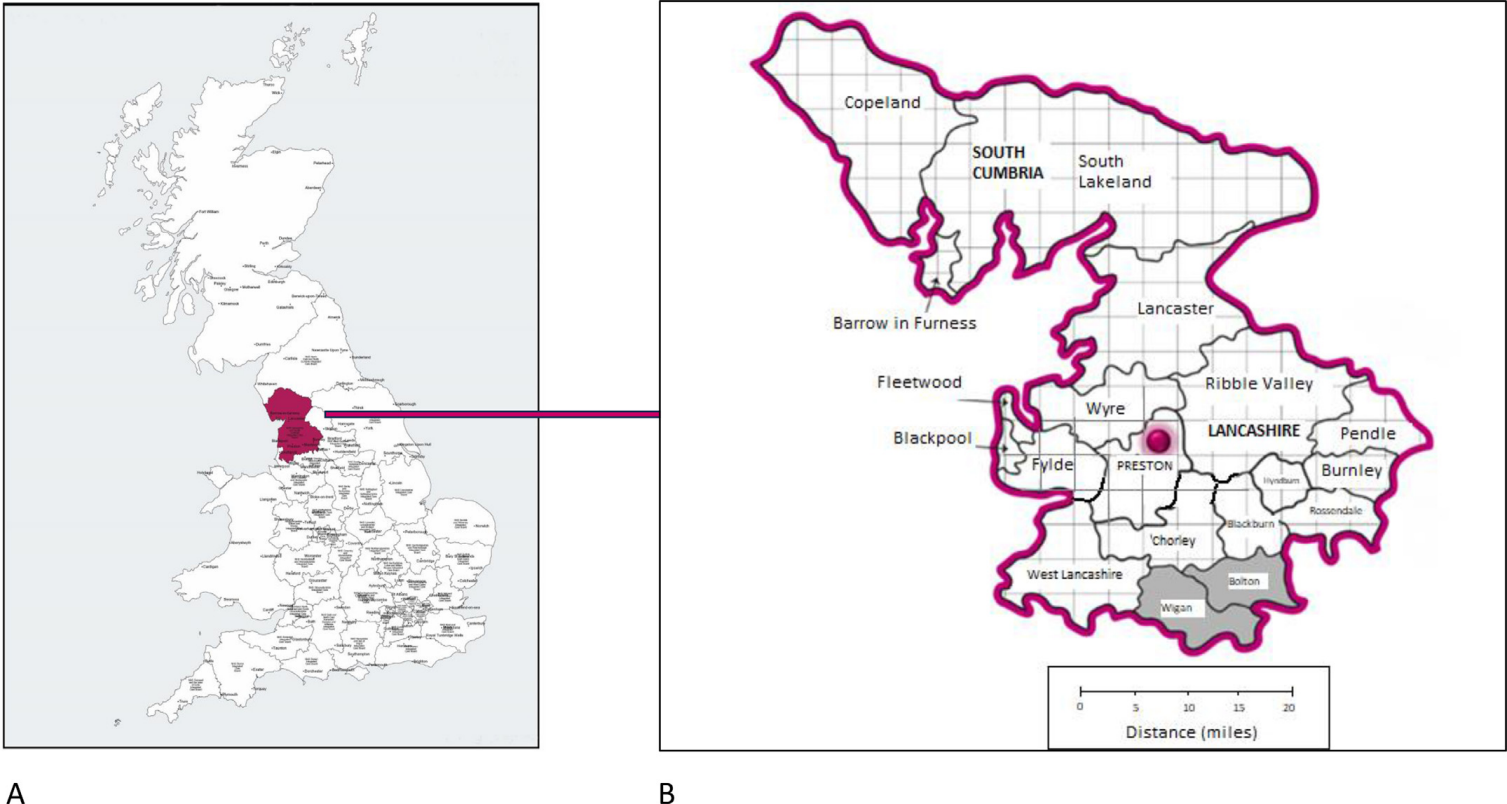
Geographical footprint of our catchment area of our renal centre in the North West of England, United Kingdom (approximation). The catchment population is around 1.5 million across some urban conurbations and rural areas. The maximum distance one of our patients could travel by car from the Northernmost point in our region to the renal hub in Preston is around 2 h (Google Maps™).

Climate change is the greatest global health threat of the 21st century.^1^ Healthcare provision is a significant contributor and accounts for 4–5% of the UK carbon footprint.^2^ Vanholder *et al.* suggested that nephrology working practices should be altered to reduce the impact on climate change.^3^ Current UK guidance suggests that uncomplicated renal transplant patients should be monitored 3–6 monthly.^4^ Royal Preston Hospital is a tertiary renal centre in North West England that looks after approximately 900 patients with a functioning transplant from a large geographical area in Lancashire and South Cumbria (Fig. 1). Traditionally, all consultant-delivered transplant aftercare in our centre was provided through face-to-face appointments mostly at the hub in Preston while nurse-led appointments were all carried out via telephone. As a result, many patients regularly travelled large distances to attend their follow up appointments, almost exclusively in fossil-fuel powered cars with significant environmental impact. However, because of the Coronavirus disease 2019 (COVID-19) pandemic, many of these appointments are now delivered virtually. We were interested to study how this development affects the environmental footprint of our transplant aftercare, compare the effect to other interventions in the field of climate change, and study financial and time savings to our patients.

## Methodology

As a result of the COVID-19 pandemic, a large proportion of routine consultations that had previously been provided through face-to-face consultation were converted to virtual appointments mostly via video through NHS Attend Anywhere™. We retrospectively evaluated the environmental impact of this development, which helped us to overcome the constraints of the COVID-19 pandemic.^5^ We evaluated a single consultant led transplant follow up clinic at the Royal Preston Hospital over a 2-year period from April 2020 to March 2022. Patients were typically beyond 6 months after transplant and stable with a preponderance of long term follow up. 68 patients were included (32 female [47.1%], 36 male [52.9%]. Age range was 26–79 years (Mean 57.9 years old. Median 61.5 years old ([QR 48–71]]. This clinic currently delivers 75% of all follow-up appointments for transplant aftercare through virtual appointments, the vast majority of which is video through NHS attend anywhere™. Only one consultant (AW) was involved in this initiative and there was no involvement of other clinicians. In stable patients, video appointments with the consultant and reviews via telephone with a transplant nurse would alternate. Immunosuppression was dispensed via a dedicated transplant pharmacy team and medication was posted out or collected during face to face appointments. Patients had their pre-clinic blood tests in local blood test clinics in the same way as before the intervention. We recorded the number of clinic appointments and whether they were delivered face to face or virtually (video or telephone) during the study period. If the clinic was virtual (video or telephone) we then calculated the miles saved, CO_2_ equivalent savings, cost savings and time savings. Commuting distances to clinic were assessed using patients’ documented address and postcode. Carbon footprint data were estimated for average size vehicles of unknown fuel source as recommended by UK government data.^6^ Cost savings were calculated using petrol prices as they were at the time of the clinic including £2.50 parking charge.^7^

## Outcome

In this period, 616 clinic appointments took place of which 416 (67.5%) were video consultations, 112 (18.2%) telephone consultations and 83 (13.5%) face to face consultations. Eventually all patients learned to carry out video consultations, some with help from relatives and occasionally only after demonstration of the concept during a face-to-face appointment. We did not record when video consultations had to be converted to telephone during the appointment, but this was an unusual event and typically prompted by poor network coverage, for example when patients were outside their homes. Face to face consultations were prompted by clinical need. Blood tests and immunosuppression monitoring were performed in the community as they were prior to the pandemic and their impact on CO_2_ emissions was not studied. Public transport was not considered given the fact that to our best of knowledge this is seldom used by our patients, partly due to the distances and the large geographical footprint of our catchment area but also because our transplant patients have become very reluctant to utilise public transport since the COVID-19 pandemic. Commute via electric vehicle was also considered negligible in this patient population during the study period.

As a result of virtual consultations (video or telephone), 42,666 travelling miles were saved, resulting in a saving of 11,718 kg CO2 emissions. This also led to savings of £8,855 in petrol and parking costs, and an estimated 540 h of patient travel time saved. Table 1 provides context for CO_2_ equivalent savings in our study, compared to other transport interventions. We did not collect systematic patient feedback, but in our experience feedback has been mainly positive. Advantages that patients typically emphasised were the convenience of attending clinics from home, avoidance of commute and time saved. Patients also reported that they liked the fact that they did not have to search for parking prior to the appointment. Older patients also felt safer from infection risks when compared to face-to-face clinics. Younger patients felt less stigmatised particularly in the workplace as the new approach allowed them to avoid taking leave for clinic appointments. Negative feedback was infrequent, often occurred early on, and related mainly to technical issues (network coverage, equipment). The positive feedback and good experience were key drivers to continue this initiative post-pandemic. We now see all stable transplant patients via video consultation and only bring them in for a face-to-face appointment where patients are not well or to discuss interventions and important decisions, such as transplant biopsy, rejection treatment, or decisions at times of transplant failure. If during a video clinic a patient is thought to benefit from face-to-face assessment then we have a portfolio of face-to-face clinics, including a pooled weekly acute transplant clinic and frequent rapid review clinics available for that purpose. We also aim to see even very stable patients face to face at least once per year.

**Table 1:** CO_2_ expenditure/savings in comparison (UK government data, in the public domain).

Intervention	CO_2_ equivalent
Petrol car journey from Glasgow to London, for a single passenger	88 kg
Flight from Glasgow to London, per passenger	157 kg
Our virtual clinic savings during the two year study period	11,718 kg
UK domestic transport emissions in 2020	99 million tonnes

We did not study clinical outcomes in detail but we have learned what is easy to miss via video when compared to face-to-face encounters: In our experience, increasing frailty and mobility issues are easily overlooked as patients are usually sat down during a video consultation. Oedema is possible to assess to some degree but difficult to quantify in a virtual encounter. Weight gain and loss are also more easily missed than in a face-to-face environment.

## Conclusion and next steps

The fact that converting appointments from face-to-face to virtual consultations led to a reduction in the associated CO2 emissions is not a surprise. What our study adds is information about the magnitude of emissions saved in a regional posttransplant service (Table 1). Our findings are in keeping with other studies which found that carbon footprint savings ranging between 0.70 and 372 kg CO_2_ per consultation can be achieved.^5^^,^^8^^,^^9^ We did not study clinical outcomes but others have reported that virtual appointments are safe and do not result in a higher rate of unplanned hospital admissions within 30 days.^10^ We did not survey patient perceptions of this service but note that others reported good patient feedback.^11^ Another study around video consultations for kidney disease care, including care of renal transplant patients, also reported high patient satisfaction, acceptable clinical outcomes and improved efficiencies.^12^

Our clinic restructure was not primarily driven by concerns around climate change, but by constraints during the global COVID-19 pandemic. We were impressed not only with the environmental impact during a short period of time, but also with the savings in time and fuel expenditure. Such savings are increasingly relevant to our patients given the cost-of-living crisis.

Our study has strengths and weaknesses. The fact that only one clinician was involved was a strength of our study, and also the fact that the approach is now well established with a stable cohort of very IT literate patients. The limitations of our study include the fact that the environmental impact elsewhere may be smaller (for example in urban centres) and the fact that transplant patients are mostly younger and IT literate whereas other clinic populations may be less suitable. In our own department, video consultations have proved to be slightly less successful and less widely used in general nephrology clinics where patients are on average older, with less IT literacy and skills. The fact that we did not ascertain the effect of commute for blood tests or use of public transport are also limitations.

Further studies are needed to assess patient satisfaction in more detail, understand barriers to virtual care, and confirm that virtual consultations deliver similar clinical outcomes. Further research is also needed to fully understand the carbon footprint of the wider posttransplant pathway, including commute to blood test clinics and delivery of immunosuppressive medication in the community.

In our experience video consultations appear safe for routine transplant aftercare and we are currently trying to evaluate this approach in other subspecialty clinics. We suggest that other transplant centres, particularly those with a large geographical footprint, should consider virtual clinics as part of their efforts around green nephrology.
